# Assessing Variation in the Individual-Level Impacts of a Multihost Pathogen

**DOI:** 10.1155/2023/4003285

**Published:** 2023-05-27

**Authors:** Zachary M. Lewin, Francisca Astorga, Luis E. Escobar, Scott Carver

**Affiliations:** ^1^Department of Biological Sciences, University of Tasmania, Hobart, Tasmania, Australia; ^2^Universidad Andres Bello, Centro de Investigación Para la Sustentabilidad, Facultad de Ciencias de la Vida, Santiago, Chile; ^3^Department of Fish and Wildlife Conservation, Virginia Tech, Blacksburg, VA, USA

## Abstract

Most pathogens infect more than one host species, and given infection, the individual-level impact they have varies among host species. Nevertheless, variation in individual-level impacts of infection remains poorly characterised. Using the impactful and host-generalist ectoparasitic mite *Sarcoptes scabiei* (causing sarcoptic mange), we assessed individual-level variation in pathogen impacts by (1) compiling all documented individual-level impacts of *S. scabiei* across free-living host species, (2) quantifying and ranking *S. scabiei* impacts among host species, and (3) evaluating factors associated with *S. scabiei* impacts. We compiled individual-level impacts of *S. scabiei* infection from 77 host species, spanning 31 different impacts, and totalling 683 individual-level impact descriptions. The most common impacts were those affecting the skin, alopecia (130 descriptions), and hyperkeratosis coverage (106). From these impacts, a standardised metric was generated for each species (average impact score (AIS) with a 0-1 range), as a proxy of pathogen virulence allowing quantitative comparison of *S. scabiei* impacts among host species while accounting for the variation in the number and types of impacts assessed. The Japanese raccoon dog (*Nyctereutes viverrinus*) was found to be the most impacted host (AIS 0.899). We applied species inclusion criteria for ranking and found more well-studied species tended to be those impacted more by *S. scabiei* (26/27 species AIS < 0.5). AIS had relatively weak relationships with predictor variables (methodological, phylogenetic, and geographic). There was a tendency for Diprotodontia, Artiodactyla, and Carnivora to be the most impacted taxa and for research to be focussed in developed regions of the world. This study is the first quantitative assessment of individual-level pathogen impacts of a multihost parasite. The proposed methodology can be applied to other multihost pathogens of public health, animal welfare, and conservation concern and enables further research to address likely causes of variation in pathogen virulence among host species.

## 1. Introduction

Most known pathogens (>60%) infect more than one host species [1–3], suggesting strong evolutionary pressures on pathogens for adapting a generalist strategy to invade multiple species via multiple mechanisms [3, 4]. Pathogens may invade different host species as a function of interspecific interactions, encounters with environmental fomites, stages of the pathogen life cycle, and as a function of being dispersed to new geographic locations [5, 6]. Anthropogenic movement, through trade, transportation, and interactions at the human-wildlife interface, is an important contributor to the number of hosts a pathogen may potentially encounter [3, 7–10]. Considering the global risks multihost pathogens pose to human health, agriculture, and biodiversity, the study of multihost pathogen impacts is important [11].

The effect that multihost pathogens have on host species varies [12–14], but the extent of variation in pathophysiological impacts (pathogen impacts) is rarely understood. In principle, there can be multiple mechanisms driving variation in pathogen impacts among host species. For example, where cross-species transmission (spillover) occurs regularly, a pathogen may evolve to have the greatest impact (i.e., virulence) on the new host species [2, 15, 16]. Host immunity also influences variation of pathogen impacts, both by mitigating the impact through defending the host against damage caused and by triggering an exaggerated self-damaging immune response (termed immune-mediated pathology or immunopathology) [17–19]. Variation in pathogen impact among hosts can also be related to tolerance to infection, such as through investment in repair mechanisms against pathogen-induced damage [20–22]. Under high tolerance to infection, a host species may maintain a relatively high prevalence of infection, while only experiencing mild disease (low pathogen impact or subclinical infection). During high tolerance to infection, a host species can serve as a pathogen reservoir facilitating pathogen spread to new species and areas [23, 24]. Pathogens can also develop host-specific strategies, which induce differing disease expressions, such as pathogens that undergo multiple life-stages between different host species, inducing life-stage specific effects among hosts [25]. In addition, variation in host behaviour is known to influence the effects a pathogen can have, such as through variation in social grooming (removing parasites) [26] or propensity to wake from hibernation [27]. Finally, variation in pathogen impacts across host species is expected in multihost panzootic and emerging diseases, as pathogens encounter novel hosts species [28].

The assessment and comparison of pathogen impacts across host species are important yet theoretically and practically challenging. Current pathogen impact assessments in animals range from descriptions of population-level impacts [29] to welfare-oriented assessments [30]. Formal comparisons of pathogen impacts have been made for several multihost pathogens in free-living animals [12–14]. However, these comparative assessments are all population based, and interhost species variation in individual-level impacts (i.e., given an infection, how impacted are individuals of a host species) has not been investigated. The variability in quality and quantity of data able to be extracted from field conditions creates challenges with quantitatively assessing individual-level pathogen impacts in free-living animals [31, 32]. Thus, the formation of metrics to assess individual-level impacts across host species is needed, particularly from an animal pathology and welfare perspective [33, 34].

*Sarcoptes scabiei*, the ectoparasitic mite which causes sarcoptic mange, has been documented in at least 148 species, across 12 orders, and 29 families, making it one of the most host-generalist pathogens infecting mammals [10]. Sarcoptic mange is both an emerged and emerging wildlife disease, having a near global distribution [10, 35], with notable highly impacted host species such as the red fox (*Vulpes vulpes*) [36], bare-nosed wombat (*Vombatus ursinus*) [37], and Iberian ibex (*Capra pyrenaica*) [38]. *Sarcoptes scabiei* also negatively impacts domesticated animals [39] and is a neglected tropical disease of humans (termed scabies in humans) [40]. Transmission of this multihost parasite spans from environmental to direct mechanisms [41]. For social host species, direct transmission is dominant, while for solitary species environmental (indirect) transmission is key [42]. Upon infecting a host, the *S. scabiei* mite burrows into the host's skin, feeding on dermal tissue and interstitial fluids [43]. The lifecycle of the mite involves five stages: egg, larva, protonymph, tritonymph, and adult, and generally takes 10–13 days [44].

*Sarcoptes scabiei* impacts are often driven by host immunopathology, such that clinical signs and expression of the disease are often a result of an exaggerated immune response to the mite infection [18]. Common signs of *S. scabiei* infection include alopecia (hair loss), hyperkeratosis (the thickening of the skin), pruritus (itching of the skin), fissuring of the epidermis, and in many species, infection can lead to emaciation and death [45, 46]. Immunopathological responses of infected hosts are generally classified as Type I or IV hypersensitivity reactions [18], although both types of hypersensitivity can occur in the same host. A Type I hypersensitivity reaction (i.e., immediate antibody-mediated immune response) is commonly associated with the less severe impact, also known as “ordinary mange,” typified by pruritus and alopecia. A Type IV hypersensitivity reaction (i.e., delayed cell-mediated immune response) causes “crusted mange,” which is the most severe impact, causing hyperkeratosis, skin crusting, and death. Thus, *S. scabiei* impact among host species varies based on the type and strength of the host immune response.

*Sarcoptes scabiei* is relatively well studied with recent reviews summarising cross-species transmission and conservation threats [10, 41]; immunological and pathological impacts [18]; treatment in free-living animals [47, 48]; and a range of taxon and region specific reviews [37, 49–54]. A critical gap that remains in the field is a synthesis of the individual-level impacts it has across host species. Given the expanding impact of *S. scabiei* in wildlife and its relevance to conservation and animal welfare, further understanding of how *S. scabiei* impacts its host species is very important. Our overarching aim is to advance understanding of individual-level variation in pathogen impacts, focusing on *S. scabiei* infection among host species. To achieve our overarching aim, we have three objectives: (1) to compile all documented individual-level impacts of *S. scabiei* infection across all free-living host species, (2) to quantify and rank the variable impact of *S. scabiei* infection across its free-living host species, and (3) to evaluate factors likely associated with *S. scabiei* impacts.

## 2. Methods

### 2.1. Individual-Level Impacts of *Sarcoptes scabiei* on Host Species

**Figure 1:**
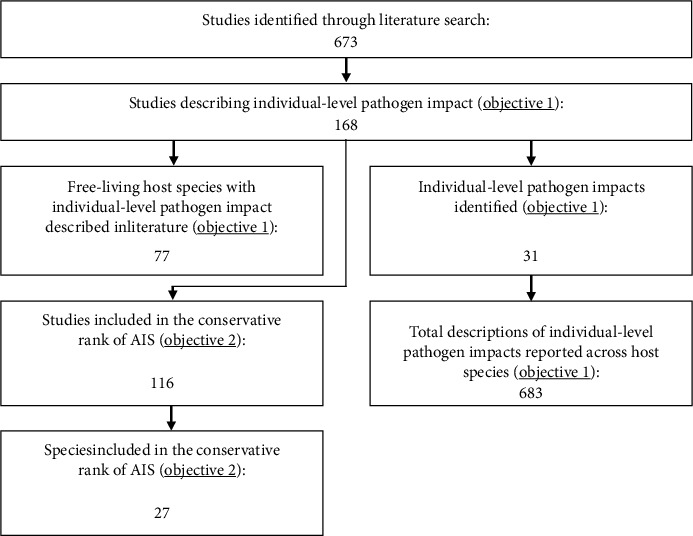
Flow diagram summarising the number of studies, *Sarcoptes scabiei* impacts, and host species identified from literature search. From the total number of studies initially compiled (673), we only included those that described individual-level *S. scabiei* impacts. From those 168, we identified 77 hosts, 31 impacts, and 683 descriptions. Further inclusion criteria were applied (see Table 2) and the number of studies (116) and host species (27) are also shown.

A systematic literature search was developed to collate all reporting of individual-level impacts from *S. scabiei* in its free-living host species. An individual-level pathogen impact was defined as a measurable effect on an individual animal's physiology or behaviour due to infection. First, we generated a list of all known susceptible hosts based on a previous comprehensive review [10], adding 10 additional host species found by the authors (see Supplementary Materials S1). To explore comparable host response to *S. scabiei* infection, we excluded humans and only included species living under free-ranging conditions (neither supervised nor dependent on humans), which included a specific inclusion criteria to define a free-living species (see Supplementary Materials S2). In the Web of Science, we used a keyword string that combines *hosts* AND *sarcoptic mange*. For hosts, we used common and scientific names including subspecies. For mange, we used “sarcoptic mange” or “*Sarcoptes scabiei*” or “mange” or “scabies.” We complemented these results with a Google Scholar search, using the host species' most accepted common name and “sarcoptic mange.” We also included additional papers found in the references within the collected papers. A total of 673 papers were collected in this literature search, which were screened for studies containing a description of an individual-level *S. scabiei* impact resulting in 168 studies (Figure 1).

*Sarcoptes scabiei* impact descriptions were extracted from these studies and collated into a database and categorised by their area of impact. Areas of impact included external manifestations (impacts affecting the skin), class effects (sex or age classes being disproportionately affected within a population), metabolic (physiological impacts that are not external or immunological), immunological (impacts affecting capacity to recover from infection), or behavioural. The total number of studies that described an individual-level impact was noted for each host species, along with the number of different impacts (31 impacts across all host species) and the total number of descriptions for each impact (683 total descriptions across all host species) (Figure 1, also see Results).

### 2.2. Quantify and Rank the Variable Impact of *Sarcoptes scabiei* across Host Species

To compare the impact caused by *S. scabiei* across its host species, we developed a combined single metric, which was standardised, quantitative, and accounted for the variation in the number and types of impacts assessed. For each impact described in a study, its host species was noted, and an *impact score* was assigned. To standardise and score all 31 impacts identified in the literature, a nominal scale of increasing effect on the host was created. The number of intervals used for each impact was determined from the detail by which the impact was generally described in the literature (see Supplementary Materials S3). *Sarcoptes scabiei* impacts regularly quantified or precisely described, such as those that assess body coverage, were assigned more interval levels, and thus captured more detail, while those that were more broadly described were allocated fewer intervals. The *impact score* always ranged from impact not observed given infection to the maximum observed effect on a host, across all host species, from that impact. Some impacts received a binary standardisation, as either present or absent owing to how they were reported in the literature (e.g., the reduction in vigilance behaviours, or the presence of anaemia). For the full list of impact criteria, quantification, and standardisation see Supplementary Materials S3. To ensure all *S. scabiei* impacts were comparable, *impact scores* were all scaled to between 0 and 1 to form the *standardised impact score*:

(1) $$\begin{array}{c} {sis}_{i}=\frac{{is}_{i}}{{is}_{\max}}\text{,} \end{array}$$

where sis_*i*_ is the *standardised impact score* for host species *i*, *is*_*i*_ represents *impact score* for host species *i*, and *is*_max_ is the maximum value that said *impact score* can be across host species.

Additional variables were recorded to capture factors that potentially influence the comparison of *S. scabiei* impact among host species. The number of animals used across studies to assess an *S. scabiei* impact for a host species was collected as the *sample size for the impact*. The range of *standardised impact score* identified within a study was also collected, as a *minimum standardised impact score* and *maximum standardised impact score*. The precision in reporting of *S. scabiei* impacts in the literature also varied greatly. For example, many descriptions of conditions were qualitative, and a level of interpretation was required to assign those descriptions into the intervals of increasing impact described above. To capture the degree of required interpretation, a *confidence in standardisation score* was created (see Supplementary Materials S4 for *confidence in standardisation score* criteria).

When multiple studies assessed the same impact for a host species, the *standardised impact scores* were averaged, using their sample sizes as a weighting factor:

(2) $$\begin{array}{c} {asis}_{i}=\frac{\sum \left(\begin{array}{c} {sis}_{ij}\times \\ n_{ij} \end{array}\right)}{N_{i}}\text{,} \end{array}$$

where asis_*i*_ is the *average standardised impact score* for host species *i*, sis_*ij*_ is the *standardised impact score* from each study *j* for each host species *i*, *n*_*ij*_ is the sample size of individuals assessed from each study *j* for each host species *i*, and *N*_*i*_ is the total sample size of individuals used from all studies to assess *S. scabiei* impact for host species *i*. The same was done with the *confidence in standardisation scores*, with *confidence in standardisation score* for each study replacing sis_*ij*_, to form *standardised confidence in the standardisation score*.

For comparison of *S. scabiei* impact across free-living host species, an *average impact score* (*AIS*_*i*_, range 0-1) was created:

(3) $$\begin{array}{c} {AIS}_{i}=\frac{\sum {asis}_{i}}{N^{0}i}\text{,} \end{array}$$

where AIS_*i*_ is calculated as the sum of *average standardised impact scores* (asis_*i*_) for host species *i*, divided by the number of different *S. scabiei* impacts recorded for host species *i* (*N*^0^*i*). The *average impact score range* was calculated using equation (3) as well, with *minimum standardised impact score* and *maximum standardised impact score* replacing asis_*i*_, to provide a range value for each AIS_*i*_. An *average confidence in standardisation score* and *average sample size per impact* was also calculated for each host species using equation (3), with *standardised confidence in standardisation scores* and *sample size for the impact* replacing asis_*i*_.

Initial ranking of AIS scores (see Supplementary Materials S5 for the full rank of host species prior to applying conservative inclusion criteria) was likely confounded owing to variation in the quality of information available for some host species. Thus, a more conservative rank was developed whose inclusion criteria aimed to ensure a greater level of confidence in assigned AIS_*i*_ values. Four variables were used to create this more conservative rank: *average sample size per impact*, *average confidence in standardisation score*, *number of pathogen impacts reported for a species*, and *number of studies assessing each species*. The inclusion criteria for a host species to remain in the conservative rank of AIS are shown in Table 1.

### 2.3. Evaluating Factors Associated with *Sarcoptes scabiei* Impacts

To evaluate factors associated with individual-level *S. scabiei* impacts, we examined a combination of predictor variables that potentially influenced the quality of the AIS. Predictor variables included *average sample size per impact*, *average confidence in standardisation score*, *number of studies assessing pathogen impacts for a species*, *number of pathogen impacts assessed for a species*, and the *proportion of impacts binary in their standardisation*. The *proportion of impacts binary in their standardisation* was included to assess if the extreme nature of binary values compared to those with more assigned nominal intervals affected AIS scores. The nonmethodological variables of the taxonomic status of host species (order) and the development status of the country where a host species was most often studied, assigned according to the UN “Developed Economies, 2022” [55], were also collected. The effect of these predictor variables on the AIS was assessed using a generalised linear model (GLM). All analyses were conducted in *R* statistical software version 4.1.2 [56], using the packages “tidyverse” [57], “Hmisc” [58], and “PerformanceAnalytics” [59].

Prior to GLM analysis, all continuous predictor variables were scaled between 0 and 1, so the effect size of the predictor variables was comparable. A Pearson correlation matrix was also used to assess covariance among all continuous predictor variables to remove correlated (*R* > 0.7) variables (see Supplementary Materials S6 for results of the Pearson correlation matrix). The *number of studies assessing pathogen impacts for a species* was correlated with multiple other variables and thus removed. All predictor variables were then assessed for their effect on AIS, represented by the equation as follows:

(4) $$\begin{array}{c} AIS\;∼\;average\;sample\;size\;per\;impact+average\;confidence\;in\;standardisation\;score\\ +number\;of\;pathogen\;impacts\;assessed\;for\;a\;species\\ +proportion\;of\;impacts\;binary\;in\;their\;standardisation\\ +developmental\;status\;of\;country\;where\;species\;is\;most\;studied\\ +host\;species\;order\text{.} \end{array}$$

## 3. Results

### 3.1. Individual-Level Impacts of *Sarcoptes scabiei* on Host Species

**Figure 2:**
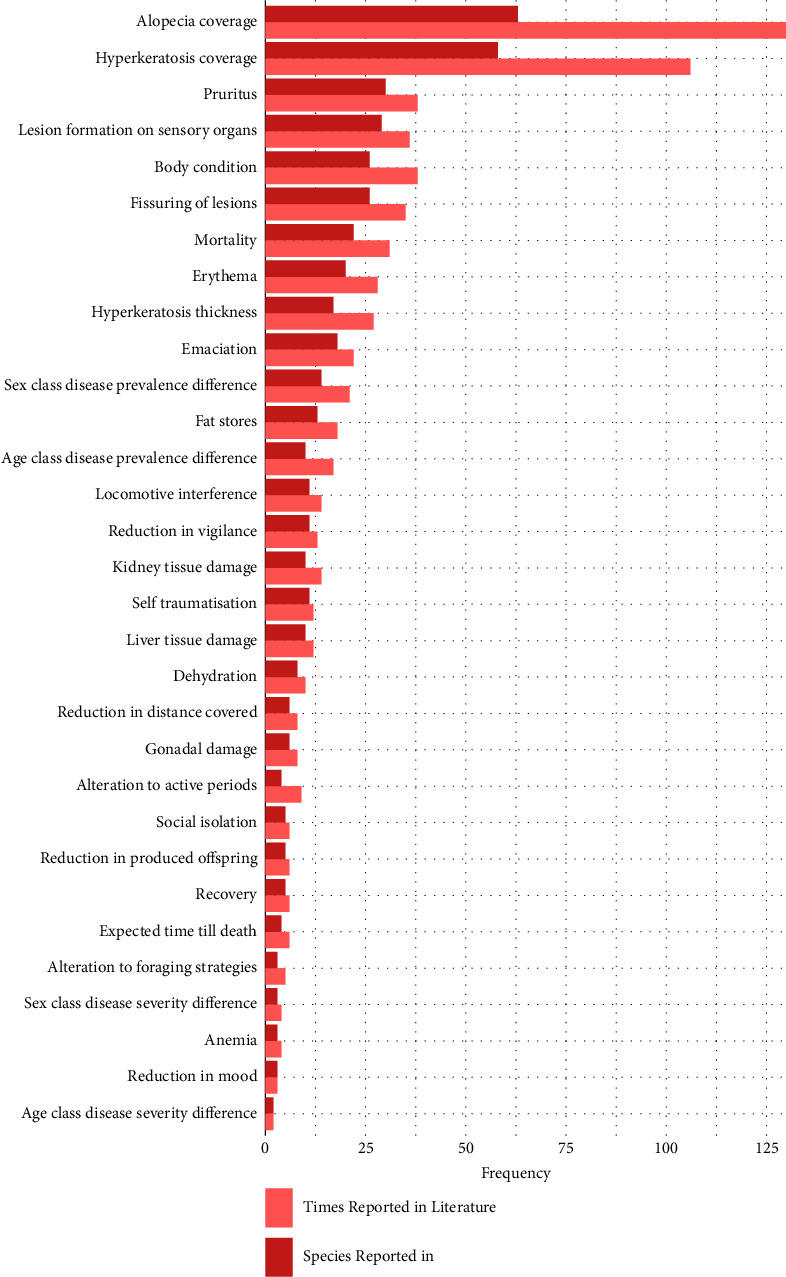
Individual-level impacts from *Sarcoptes scabiei* plotted against the number of species assessed by each impact within the literature (dark red), and total number of times reported within the literature (light red). Impacts are sorted according to the sum of both variables.

**Figure 3:**
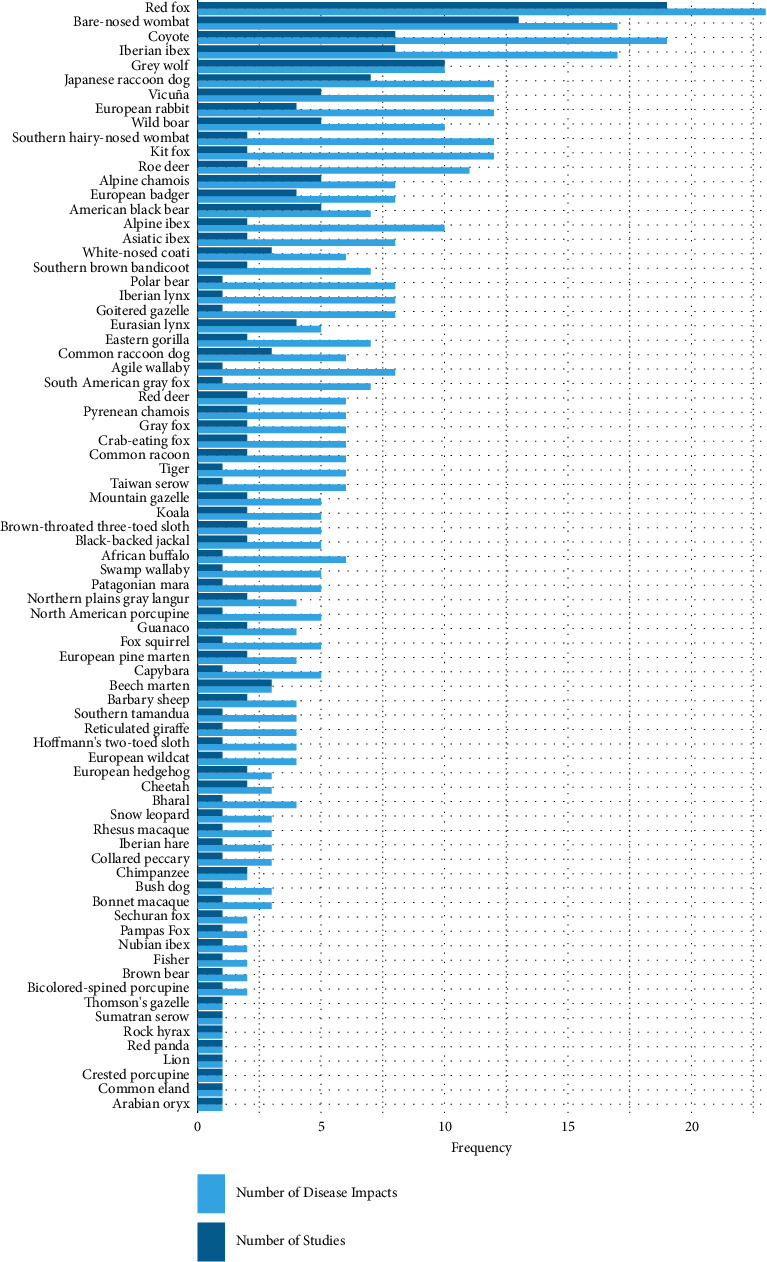
Number of studies assessing individual-level *Sarcoptes scabiei* impacts. Dark blue: denotes the number of studies. Light blue: denotes the number of *S. scabiei* impacts assessed within the literature for each free-living host species. The species are sorted according to the sum of both variables.

A total of 31 individual-level pathogen impacts were identified across 77 free-living host species within the literature, with a total of 683 descriptions reported (Figure 1). The types of *S. scabiei* impacts described the most were external manifestations (impacts affecting the skin) reported 415 times (59.6% of total reportings), followed by metabolic impacts (nondermal or immunological and physiological impacts) reported 167 times (24.0%) (Table 2). Of the individual-level pathogen impacts extracted, alopecia coverage (130) and hyperkeratosis coverage (106) were reported most often and assessed across most host species (63 and 58, respectively) (Figure 2). The free-living host species with most studies assessing individual-level *S. scabiei* impacts were the red fox (19), the bare-nosed wombat (13), and the grey wolf (10) (Figure 3). The host species with the most individual-level *S. scabiei* impacts assessed within the literature were the red fox (23), coyote (*Canis latrans*) (19), bare-nosed wombat (17), and Iberian ibex (17).

### 3.2. Quantify and Rank the Variable Impact of *Sarcoptes scabiei* across Host Species

**Figure 4:**
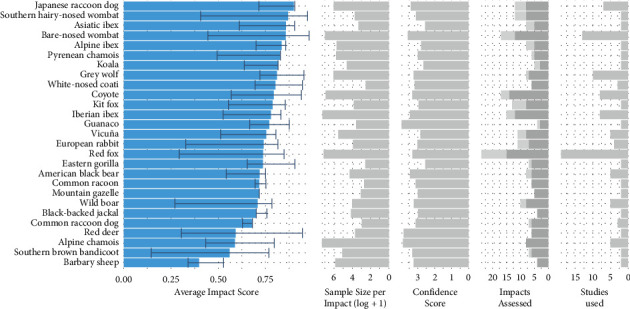
Free-living *Sarcoptes scabiei* host species (27) plotted against the average impact score (AIS) for *S. scabiei* impacts, with conservative criteria applied and the average range of AIS also shown. Plotted to the right are variables used in the creation of the conservative rank (cf. Table 1): average sample size per Impact (log + 1), average confidence in standardisation per impact scores, total *S. scabiei* impacts assessed per species (in light grey, impacts that were standardised into binary data, and in dark grey, impacts standardised into interval data), and studies used per species.

The conservative rank of the average impact score (AIS) which allows for comparison of *S. scabiei* impacts among host species can be seen in Figure 4 (also see Table 1 for conservative inclusion criteria and Supplementary Materials S7 for visualisation of cut off points). The species with the highest AIS was the Japanese raccoon dog (*Nyctereutes viverrinus*), with a score of 0.899 followed by the southern hairy-nosed wombat (*Lasiorhinus latifrons*) (0.869), the Asiatic ibex (*Capra sibirica*) (0.858), and the bare-nosed wombat (0.857). The AIS range (shown in error bars) showed overlap in the range among host species. All host species in the conservative rank exhibited relatively high impact scores (AIS > 0.5), except for the Barbary sheep (*Ammotragus lervia*) (0.399) (Figure 4).

### 3.3. Evaluating Factors Associated with *Sarcoptes scabiei* Impacts

**Figure 5:**
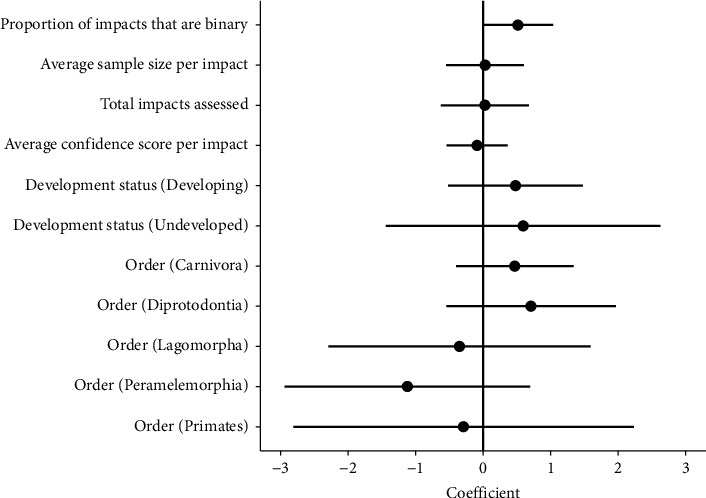
Coefficient plots from generalised linear model showing the relative impact each variable has on the average impact score for *Sarcoptes scabiei* on host species from the conservative rank (Figure 4). Continuous predictor variables are used to form the conservative AIS rank, proportion of impacts that are binary, average sample size per impact, total impacts assessed, and average confidence in standardisation per impact score. Taxonomic order of host species are shown as well, with intercept as Artiodactyla. Development status [55] of countries where host species is most studied are shown too; intercept is developed.

**Figure 6:**
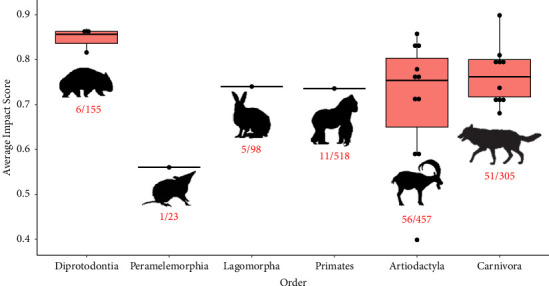
Box and whisker plot of host species from the conservative average impact score rank (Figure 4) organised into their taxonomic order plotted against average impact score for *Sarcoptes scabiei*. The numbers beneath each plot represent the number of species in each order known to be infected by *S. scabiei* out of the total number of species within each order (see Supplementary Materials S8). Analyses organised into taxonomic family can be seen in Supplementary Materials S9 and S10.

**Figure 7:**
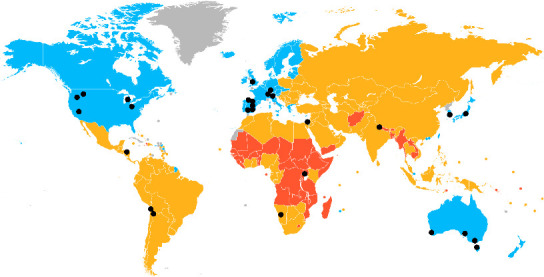
Representative location of studies used to assess impacts of *Sarcoptes scabiei* host species used in this study across the globe. Map is coloured by the development status of nations according to the UN “Developed Economies, 2022” [55]; “IMF advanced economies and UN least developed countries” by Allice Hunter is licenced under CCO 1.0; blue represents developed countries, yellow represents developing countries, orange represents undeveloped countries, and grey represents data unavailable. Black dots represent where each host species within the conservative AIS rank (Figure 4) has been most studied.

Overall, the assessed predictor variables had relatively weak relationships with the AIS (Figure 5). The *proportion of impacts binary in their standardisation* had the greatest influence in the GLM, as observed by the 95% CIs only slightly overlapping zero (Figure 5). The order Artiodactyla (even-toed hooved mammals) and Carnivora (main carnivorous order of mammals) composed the majority of host species from which a conservative AIS was calculated (77%). The average AIS among host orders did not differ significantly from Artiodactyla (Figure 5). Although not significant, there was a trend for Diprotodont, Carnivoran, and Artiodactyla species to exhibit higher average AIS values than species from the Primates, Lagomorpha, and Peramelemorphia (Figure 6). We also found a country's development status had no notable association with the AIS (Figure 5). Nevertheless, most host species making the conservative AIS rank generally came from developed countries, particularly the US, Europe, and Australia (21 of 27 species in the conservative AIS rank originating from developed countries). Capacity to detect a relationship may therefore be influenced by research primarily coming from more developed countries (Figure 7).

## 4. Discussion

While variable population-level impacts of pathogens among host species have been studied for several pathogens [12–14], variation in the individual-level impacts given an infection is poorly understood. To address this gap, we generated a standardised metric (*average impact score*, AIS) that enables the comparison of pathogen impacts across hosts, representing a novel framework to understand the individual-level impacts of multihost pathogens such as *S. scabiei.* Given an *S. scabiei* infection, we show that pathogen impacts affecting the skin are most well studied, namely alopecia and hyperkeratosis. We also found that *S. scabiei* has a wide range of impacts on host species, although this range narrowed and focused on more impacted species when using more restrictive study inclusion criteria, suggesting more well-studied hosts are also more impacted. We found Diprotodontia (Australian herbivorous marsupials) generally had the highest average AIS among host orders, and a bias for research to be focused on higher impacted species and more developed regions of the world. This study is the first to evaluate the individual-level impacts of a generalist pathogen across a diverse host range. Findings are expected to advance our understanding of the impact *S. scabiei* has among its host species and provide a framework to assess pathogen impact variation for other multihost pathogens.

### 4.1. Individual-Level Impacts of *Sarcoptes scabiei* on Host Species

A notable aspect of the literature in this study is the occurrence of research themes in pathogen impacts assessed among host species, especially for well-studied host species. For example, red foxes were commonly used to assess population and community-level effects of infection [60–62]. In contrast, studies on bare-nosed wombats, Iberian ibexes, and Japanese raccoon dogs were often used to assess the immunological and physiological effects given infection [38, 63–69], whereas coyotes and grey wolves were more frequently used to assess the behavioural and social effects of infection [70–73]. Despite these themes, there were also significant consistencies among studies, such as the overwhelming focus on alopecia and hyperkeratosis among host species, reflecting the widespread use of these variables to classify host species infected with *S. scabiei* and also define impact severity [69, 74–76]. These consistencies among host species enabled sufficient interspecific comparisons, however higher degrees of standardisation in assessing pathogen impacts among host species, such as the standardised clinical scales applied in body condition and respiratory diseases within veterinarian science [77, 78], would enable improved comparative insight into the relationships that underpin pathogen impact variation for multihost pathogens.

There were also general research areas in need of greater attention within the literature, such as the behavioural impacts of *S. scabiei* infection, despite clear impacts shown among the few studies that do tackle that topic. For example, bare-nosed wombats alter their nocturnal foraging strategy to emerge from their burrows earlier and forage longer due to the metabolic and thermoregulation impacts of *S. scabiei* infection [69, 79]. Similarly, *S. scabiei* infection also altered coyote behaviour, resulting in them preferring a more scavenging mode of foraging, with increased occupation of human-built areas [71, 80]. Amlberg et al. [72] found that the risk of mortality for an *S. scabiei*-infected wolf decreased with pack size, and Cross et al. [73] showed that daily wolf movement decreased with infection severity. Further investigation into the behavioural impacts of *S. scabiei* infection on host species (e.g., change in foraging strategies, sexual behaviours, or use of environment) would be of value to provide a richer set of insights into the effects this pathogen can impose on host species.

### 4.2. Quantifying and Ranking the Impact of *Sarcoptes scabiei* across Host Species

A previous challenge to achieving assessment of individual-level variation in pathogen impacts has been the variation in the number and types of impacts assessed across host species. We overcame this variation by creating a metric that allowed interspecific comparison of host impacts—the AIS. The AIS developed in this study is elegant in its simplicity, accounts for variation in pathogen impacts measured across host species, and allows for objective comparison among host species. As the AIS operates as an average of the relative impact that a pathogen has on its host species, it can also act as a possible measure of pathogen virulence across host species. For sarcoptic mange, this has a value for explorations of the factors underscoring why some host species are impacted more than others from an infection. However, the value of the AIS is also broader. Given the significant threat that other multihost pathogen systems have on biodiversity, human health, agriculture, and animal welfare [11], the AIS metric could be readily applied to other multihost pathogen systems. For example, chytridiomycosis, toxoplasmosis, white-nosed syndrome, canine distemper virus, and rabies could all be assessed using the approach developed in this study. Applying a similar process to assessing pathogen impacts in other multihost pathogen systems could be particularly valuable for disentangling the patterns and causes of disease severity among host species and directing research to support taxa experiencing the greatest impact.

The conservative AIS ranking provided interesting insights into the well-studied host species of *S. scabiei*. Given that the variation observed in *S. scabiei* impact is expected to result from differences in the host's immune-mediated response to infection [18]; comparison of AIS values can inform these immunological differences among host species. It was unsurprising that the Japanese raccoon dog was the highest impacted species in the conservative AIS ranking (0.889), given the severity of *S. scabiei* infection often described in this species. However, its closest relative, the common raccoon dog (*Nyctereutes procyonoides*) was ranked much lower (0.681), which suggests that despite their close genetic relatedness [81], these two species express quite different immune-mediated responses to *S. scabiei* infection. In contrast, the two wombat species in the ranking, the southern hairy-nosed wombat and the bare-nosed wombat, were relatively closely ranked (0.869 and 0.857, respectively) with very similar AIS ranges (0.406–0.973 and 0.445–0.981, respectively). This suggests that given *S. scabiei* infection, these hosts experience a similar immune reaction, characterised by Type IV hypersensitivity-driven immunopathology [37, 63, 82, 83]. The raccoon dogs and wombats exemplify that there is variation in the immune-mediated response to infection between closely related species, and that taxonomy may not be sufficient on its own to predict *S. scabiei* impact, which is supported by our comparison of host orders. A point of interest for the wombats is that the epidemiological nature of sarcoptic mange is quite different between the two species [37]. *Sarcoptes scabiei* is present throughout most of the bare-nosed wombat's range, with multiple population declines observed [84–86], whereas *S. scabiei* is scarcely present in the southern hairy-nosed wombat's range [87], illustrating the importance of not extending individual-level impacts to population impacts. The red fox was ranked towards the middle of the conservative AIS ranking (0.737), indicating that the immunopathology associated with its *S. scabiei* infection is not as severe on average as some other host species, yet it has received the most attention within the literature. The red fox has experienced occasional marked population declines throughout much of its range due to sarcoptic mange, with the disease persisting in relatively high prevalence [62, 88]. This again suggests that individual response to infection (immune-mediated pathology) may not fully explain the population-level effects of sarcoptic mange.

The AIS ranking also provided insights into both the expression of infection in species and patterns of *S. scabiei* host research. The AIS range represents the intraspecific variation seen in disease expression for a host species. This is meaningful as it can tell us if an *S. scabiei* infection in species generally leads to a similar impact in a species given infection, or if individual variation plays an important role. Some species like the southern brown bandicoot (*Isoodon obesulus*) showed large variation in the AIS range (0.145–0.767), while other species, such as the common raccoon dog, show a narrower variation (0.628–0.681). The minimum measure of the pathogen impact is susceptible to skew from observations taking place during the early stages of disease progression in an animal, and thus we were careful not to overinterpret range values for each host species. The only way to control for range values is through longitudinal studies of an individual's infection, which is difficult in free-living animals, and thus rare in the literature. The well-studied *S. scabiei* hosts, which met the criteria for the conservative AIS rank, were almost all highly impacted (AIS < 0.5). This represented a bias within the literature for well-studied species to also be those worse affected by *S. scabiei* infection. Understanding the individual-level impacts of *S. scabiei* on lesser impacted species would be valuable to enable a more robust exploration into the factors that may influence pathogen impacts. Knowing which factors are shared among lesser impacted hosts can be just as informative as knowing which factors are shared among highly impacted hosts.

### 4.3. Evaluating Factors Associated with *Sarcoptes scabiei* Impacts

We looked at several factors considered to possibly shape the AIS. Some were methodological and others were taxonomic and geographic. We found that the methodological factors had a relatively weak influence on the AIS. However, the *proportion of impacts binary in their standardisation* had a stronger effect on AIS than any other predictors. We acknowledge that binary variables may disproportionately impact the AIS owing to the extreme nature of those values relative to continuous variables. These variables were standardised to binary values due to the lack of details described in the reporting of these impacts, or the nature of their impact (either present or absent). To account for the effect that binary values had on the AIS, all impacts were scaled to the same 0-1 range, yet for further applications of the AIS, pathogen impact descriptions should preferably contain sufficient detail to assign more nominal intervals during the standardisation process.

The development status of the country where a species was most often studied [55] was not statistically associated with the AIS. However, species that were well studied, in the conservative AIS rank, tended to be from developed countries (21 of 27). This highlights a bias of research effort where a species' proximity to investigators with better funding resources may influence whether it receives research or management attention rather than just the severity of its *S. scabiei* infection. This pattern is likely seen throughout many areas of science, yet it is still important to note that there are likely highly impacted host species of *S. scabiei* in developing and undeveloped regions that are relatively understudied. For example, there have been multiple reports of severe infections and population declines due to sarcoptic mange in host species from less developed regions, such as rock hyraxes (*Procavia capensis*) [89], bicoloured-spined porcupines (*Coendou bicolour*) [90], and northern plains grey langurs (*Semnopithecus entellus*) [91], but there were insufficient data to include them in the conservative AIS ranking.

The Diprotodonts were the highest impacted taxonomic order on average (AIS = 0.847). Members of this order in the conservative AIS ranking included the bare-nosed wombat, southern hairy-nosed wombat, and the koala (*Phascolarctos cinereus*) (all members of the suborder Vombatiformes), who all display severe impacts given an infection. The orders Artiodactyla and Carnivora made up the majority of species in the conservative AIS ranking (77%), which mostly consisted of the subfamily Caprinae (goat-like antelopes) and the family Canidae (dogs and foxes) with seven species each. *Sarcoptes scabiei* is known to infect a large proportion of these orders, with 56/457 Artiodactyls and 51/305 Carnivorans reported as hosts [92] (see Supplementary Materials S7 for an overview of mammalian orders known to be infected by *S. scabiei*). Research into variable immune-mediated pathology in these orders would be valuable for understanding their comparative variation in susceptibility to *S. scabiei* impacts.

The AIS rank has potential animal welfare consequences as well. Welfare in free-living animals is socially significant yet is very difficult to assess [93, 94]. Established methods used to assess animal welfare are framed around domesticated animals [95], and the assumptions used in these models are generally not suited to the welfare impediments faced by free-living species [34, 96]. Animal welfare methods are also qualitative in nature, and thus their application in comparison among species and analysis of predictive factors is challenging. Nevertheless, the AIS has potential to act as a step toward quantitatively assessing the welfare impairment experienced by free-living animals from diseases.

While not directly comparable to this study, it is important to acknowledge other methodologies used to summarise pathogen impacts on hosts which also relate to welfare. An important one of those is disability-adjusted life years (DALY), used in human health to quantify and compare the impact of diseases or health conditions on populations using a summary measure of both mortality and disability induced by diseases [97]. More recently, a modification of the DALY has been proposed that attempts to tackle welfare in domesticated dogs, the welfare-adjusted life years (WALY), which acted as the first study to quantify the individual-level impacts of a pathogen in nonhuman animals [98]. The WALY quantified each welfare impediment on a scale of 0 to 1, much like this study did, however, they weighted their welfare impediments in terms of how much it impacted the people's perceptions of an animal's welfare, which is quite subjective. The DALY also weights the disability of a pathogen impact. The weighting of impacts is done using patient (only in the case of humans) and expert (medical profession) opinions on how much an impact impairs an individual's welfare or health state. The higher the quality of information, the more accurate these weights can be. In the DALY, confident disability weights can be formed, as human physiology is relatively very well understood, and firsthand opinions from patients can be provided. In the WALY, these weights become subjective, as animals cannot be asked how their welfare or health state is, so deciding the relative weight of an impact relies only on veterinary interpretation. The AIS did not provide a weight factor for each impact's effect on animal health, as we believed this would be too subjective for free-living animals, as the quality of information on these species is generally much lower than in humans and domesticated animals. Thus, an average of all pathogen impacts was used instead and is a quantitatively more objective and robust approach.

## 5. Conclusions

In this study, we provided a methodology for quantifying the variable impacts a multihost pathogen has among its host species. Through the creation of the AIS, this study supports further research to investigate potential mechanisms driving variation in *S. scabiei* impacts among host species. Potential factors to investigate include measures of host immune-mediated responses, life-history traits such as the sociality of host species, detailed phylogenetic analyses, environmental factors such as climate across host ranges, and anthropogenic interactions such as contact with livestock or humans. Uncovering drivers of why some host species are more impacted than others could also help predict how novel species may be affected by *S. scabiei*. Finally, this study points to the need for a greater research effort on *S. scabiei* impacts in less developed regions of the world (e.g., South America, Asia, and Africa) where the impacts of *S. scabiei* are likely significant, but poorly understood.

## Figures and Tables

**Table 1:** Inclusion criteria for host species to remain in the conservative rank of average impact score for *Sarcoptes scabiei*, with justification for cut off given (see also S6).

Inclusion criteria variables (per host species)	Inclusion threshold	Justification
Average sample size per impact	≥4	Studies with very few individuals tend to focus on more severely impacted individuals, ≥4 sample size assessments captured a broader range of disease in a species
Average confidence in standardisation score (see S3)	≥2.5	This cut off represented a midpoint between near full confidence (3) and a moderate amount of interpretation required (2), see S3
Number of pathogen impacts assessed for host species	≥4	Host species with too few impacts assessed could skew the AIS. ≥4 impacts was deemed reasonable to gauge average impact
Studies assessing a pathogen impact	≥2	To minimise biases that may be created by relying on too few studies to calculate the AIS, ≥2 studies on a host species were required

**Table 2:** Overview summary of the types of individual-level pathogen impacts reported for *Sarcoptes scabiei* infection in free-living host species, sorted into categories of impacts. Percentages are shown in parentheses.

	External manifestations	Class ratio effects^†^	Metabolic	Immunological	Behavioural
Pathogen impacts	8 (25.8)	5 (16.1)	10 (32.3)	1 (3.2)	7 (22.6)
Times reported	415 (59.6)	50 (7.2)	167 (24.0)	6 (0.9)	58 (8.3)

^†^Refers to classes within a population (sex and age) being disproportionately affected by sarcoptic mange.

## Data Availability

The data used to support the findings of this study have been deposited in the UTAS Research Data Portal repository (https://doi.org/10.25959/12q1-g388).

## Supplementary Materials

Supplementary Materials S1: Excel file containing data frames used in data collection and analysis for this project, as well as updated host species database for *Sarcoptes scabiei*. These data are deposited in the UTAS Research Data Portal repository (https://doi.org/10.25959/12q1-g388). S2: species inclusion criteria for the study. Table S3: the grading criteria used, and the assigned number of intervals, for quantifying and standardising each individual-level pathogen impact that formed the AIS. Table S4: confidence in standardisation criteria: required interpretation needed to quantify pathogen impacts from the literature. Figure S5: the initial rank of host species plotted against the average impact score (AIS), with the AIS range shown in error bars. Plotted to the right: average sample size per impact (log + 1), total pathogen impacts assessed per species (in light grey, impacts that were standardised into binary data, and in dark grey, impacts standardised into interval data). Table S6: Pearson correlation matrix for continuous predictor variables is used in regression analysis. Significant values (*R* > 0.7) are in bold. Figure S7: diagnostic plots for inclusion cut offs (vertical red lines) for the conservative rank of *Sarcoptes scabiei* impacts among host species, showing average impact score for all host species in initial rank (77) against (A) average sample size per impact (log + 1) (with cut off at 4), (B) average confidence in standardisation per impact scores (with cut off at 2.5), (C) total pathogen impacts assessed per species (with cut off at 4), and (D) studies used per species (with cut off at 2), refer to Table 1 for justification of cut off values. Table S8: the table showing the proportion of families and species known to be infected by *Sarcoptes scabiei* for each affected order. Figure S9: boxplot of host species from conservative AIS rank organised into their taxonomic family plotted against average impact score. Red numbers represent the number of species in each family known to be infected by *Sarcoptes scabiei,* out of the number of species within each family (see Supplementary Materials S10). Table S10: the table showing the proportion of species known to be infected by *Sarcoptes scabiei* for each affected family.
