# Supplementary material for: Assessing Variation in the Individual-Level Impacts of a Multihost Pathogen
Source: Transbound Emerg Dis. 2023 May 27;2023:4003285. doi: 10.1155/2023/4003285 (PMC12017245; doi:10.1155/2023/4003285)
Supplement: Supplementary Materials — S1: Excel file containing data frames used in data collection and analysis for this project, as well as updated host species database for Sarcoptes scabiei. These data are deposited in the UTAS Research Data Portal repository (https://doi.org/10.25959/12q1-g388). S2: species inclusion criteria for the study. Table S3: the grading criteria used, and the assigned number of intervals, for quantifying and standardising each individual-level pathogen impact that formed the AIS. Table S4: confidence in standardisation criteria: required interpretation needed to quantify pathogen impacts from the literature. Figure S5: the initial rank of host species plotted against the average impact score (AIS), with the AIS range shown in error bars. Plotted to the right: average sample size per impact (log + 1), total pathogen impacts assessed per species (in light grey, impacts that were standardised into binary data, and in dark grey, impacts standardised into interval data). Table S6: Pearson correlation matrix for continuous predictor variables is used in regression analysis. Significant values (R > 0.7) are in bold. Figure S7: diagnostic plots for inclusion cut offs (vertical red lines) for the conservative rank of Sarcoptes scabiei impacts among host species, showing average impact score for all host species in initial rank (77) against (A) average sample size per impact (log + 1) (with cut off at 4), (B) average confidence in standardisation per impact scores (with cut off at 2.5), (C) total pathogen impacts assessed per species (with cut off at 4), and (D) studies used per species (with cut off at 2), refer to Table 1 for justification of cut off values. Table S8: the table showing the proportion of families and species known to be infected by Sarcoptes scabiei for each affected order. Figure S9: boxplot of host species from conservative AIS rank organised into their taxonomic family plotted against average impact score. Red numbers represent the number of species in each fami [file 4003285.f1.zip › Supplementary Material 2 (1).docx]

**S2.** Species inclusion criteria

Only free-living species were included in this study, as comparison between captive and free-living animals can become difficult for factors that influence pathogen impact. The criteria used for a free-living species was a species that contained a free-living, native population. For example, wild populations of cattle (*Bos taurus*) and domesticated goats (*Capra hircus*) exist, but they are not native, as the whole species has been domesticated. However, there are native, wild populations of pig (*Sus scrofa*) (European wild boar), and thus, European wild boar were included in this study. Care was then taken to only include studies that included the wild counterparts of these species. Even though many studies that assessed sarcoptic mange in free-living animals classified their host to the sub-species scale, the species scale was used throughout this study to provide consistency and allow greater sample sizes. This means that variation that exists between populations within a species will not be captured in our data. Species that express large phenotypic and lifestyle variation, such as *Canis lupus* which includes all wolf subspecies and dingoes, will just be counted as one host species. When uncertainty existed regarding the classification of a host species within the literature (whether they are a subspecies or their own species), we used the most recent phylogenetic papers to confirm their classification. In some cases, this meant using a different classification to the studies we was collecting data from. This particularly applied to the raccoon dogs (*Nyctereutes viverrinus* and *Nyctereutes procyonoides*), who have two recognised species, although many papers used for this to form the AIS, classified them as one species.
