# Supplementary material for: Assessing Variation in the Individual-Level Impacts of a Multihost Pathogen
Source: Transbound Emerg Dis. 2023 May 27;2023:4003285. doi: 10.1155/2023/4003285 (PMC12017245; doi:10.1155/2023/4003285)
Supplement: Supplementary Materials — S1: Excel file containing data frames used in data collection and analysis for this project, as well as updated host species database for Sarcoptes scabiei. These data are deposited in the UTAS Research Data Portal repository (https://doi.org/10.25959/12q1-g388). S2: species inclusion criteria for the study. Table S3: the grading criteria used, and the assigned number of intervals, for quantifying and standardising each individual-level pathogen impact that formed the AIS. Table S4: confidence in standardisation criteria: required interpretation needed to quantify pathogen impacts from the literature. Figure S5: the initial rank of host species plotted against the average impact score (AIS), with the AIS range shown in error bars. Plotted to the right: average sample size per impact (log + 1), total pathogen impacts assessed per species (in light grey, impacts that were standardised into binary data, and in dark grey, impacts standardised into interval data). Table S6: Pearson correlation matrix for continuous predictor variables is used in regression analysis. Significant values (R > 0.7) are in bold. Figure S7: diagnostic plots for inclusion cut offs (vertical red lines) for the conservative rank of Sarcoptes scabiei impacts among host species, showing average impact score for all host species in initial rank (77) against (A) average sample size per impact (log + 1) (with cut off at 4), (B) average confidence in standardisation per impact scores (with cut off at 2.5), (C) total pathogen impacts assessed per species (with cut off at 4), and (D) studies used per species (with cut off at 2), refer to Table 1 for justification of cut off values. Table S8: the table showing the proportion of families and species known to be infected by Sarcoptes scabiei for each affected order. Figure S9: boxplot of host species from conservative AIS rank organised into their taxonomic family plotted against average impact score. Red numbers represent the number of species in each fami [file 4003285.f1.zip › Supplementary Material 3 (1).docx]

**S3**. The grading criteria used, and the assigned number of intervals, for quantifying and standardising each individual-level pathogen impact that formed the AIS.

| **Pathogen Impact** | **Intervals assigned** | **Grading criteria** |
| --- | --- | --- |
| Alopecia | **5** | Grading of hair loss based on coverage of animal's body. (0 (none) 1 (0-10%) 2 (11-20%) 3 (21-45%) 4 (>45%)) |
| Erythema | 4 | Coverage of erythema on animal's body. (0 (none) 1 (present) 2 (affecting <45% of the body) 3 (affecting >45% of the body)) |
| Self-traumatisation | 4 | Severity of self-traumatisation. (0 (none) 1 (minimal) 2 (moderate) 3 (severe)) |
| Pruritis | 3 | Extend of starching observed. (0 (none) 1 (itching observed) 2 (animal constantly itching)) |
| Fissuring | 4 | Extent of lesion fissuring. (0 (none) 1 (present) 2 (multiple fissures present) 3 (multiple fissures of significant size present)) |
| Hyperkeratosis coverage | 5 | Coverage of para/hyperkeratosis across body surface. (0 (none) 1 (0-10%) 2 (10-20%) 3 (20-45%) 4 (>45%)) |
| Hyperkeratosis thickness | 4 | Thickness of para/hyperkeratosis. (0 (none) 1 (<5mm) 2 (5-10mm) 3 (>10mm)) |
| Body condition | 4 | Standardised body condition scores. (0 (very good, animal of healthy weight and no protruding bones) 1 (good, animal slightly underweight) 2 (poor, underweight, and signs of protruding bones) 3 (very poor, bones protruding, obvious muscle atrophy and body weight much lower than a healthy individual)) |
| Fat stores | 4 | Standardised fat reserve measure, collated from kidney, back and subcutaneous fat measures. (0 (not depleted) 1 (slightly reduced) 2 (largely reduced) 3 (almost completely depleted)) |
| Kidney damage | 4 | Severity of damage to kidney. (0 (no damage) 1 (slight amount of damage) 2 (moderate amount of damage) 3 (high amounts of damage)) |
| Liver damage | 4 | Severity of damage to liver. (0 (no damage) 1 (slight amount of damage) 2 (moderate amount of damage) 3 (high amounts of damage)) |
| Anaemia | 2 | Presence of anaemia. (0 (absent given infection) 1 (present)) |
| Emaciation | 2 | Observation of emaciation in animal. (0 (absent given infection) 1 (present)) |
| Dehydration | 2 | Observation of dehydration in animal. (0 (absent given infection) 1 (present)) |
| Locomotive interference | 2 | Reduced locomotive capacity due to infection. (0 (absent given infection) 1 (present)) |
| Reduced distance covered | 5 | Reduction to distance covered in a reported period given infection. (0 (none) 1 (0-15%) 2 (15-25%) 3 (25-50%) 4 (>50%)) |
| Reduced vigilance | 2 | Reduced awareness and avoidance of potential threats due to infection. (0 (absent given infection) 1 (present)) |
| Formation of lesions on sensory organs | 2 | Lesions on sensory organs due to infection. (0 (absent given infection) 1 (present)) |
| Alteration to active periods | 4 | Change in active periods (i.e., nocturnal or diurnal). (0 (no change) 1 (slight alteration) 2 (moderate alteration) 3 (dramatic alteration)) |
| Alteration to foraging strategies | 4 | Alteration of foraging strategies expected for species. (0 (no change) 1 (slight alteration to foraging behaviour) 2 (forages in either a different location or with a different strategy than expected for species) 3 (forages in both a different location and different strategy expected for species)) |
| Social isolation | 2 | Animal isolated from group (if applied to species). (0 (absent given infection) 1 (present)) |
| Reduction in mood | 2 | Animal described as depressed or anxious due to infection. (0 (absent given infection) 1 (present)) |
| Recovery | Continuous | Proportion of infected that naturally recovered from sarcoptic mange. (All reporting was already quantified) |
| Mortality | Continuous | Proportion of infected that die due to sarcoptic mange. (All reporting was already quantified) |
| Time till death | Continuous | Average days till death from mange infection. (Equation used to create impact score =1-((Days – 20) / 170), less than 20 days is 1, greater than 190 days is 0) |
| Reduced gonad size | 2 | Gonad (testes or ovary) reduced in size. (0 (absent given infection) 1 (present)) |
| Reduction in offspring produced | 2 | Reduction in size of litter, and frequency of reproductive events. (0 (absent given infection) 1 (present)) |
| Age prevalence differences | 5 | Age class prevalence differences (young: old). (1 (>1.5:1) 2 (1.5-1.25:1) 3 (1.25- 0.75:1) 4 (0.75-0.5:1) 5(<0.5:1))  Note: when standardising, 3 was treated as 0 point, and 1 and 5 were treated a 1 (highest impact). |
| Sex prevalence differences | 5 | Sex class prevalence differences (male: female). (1 (>1.5:1) 2 (1.5-1.25:1) 3 (1.25- 0.75:1) 4 (0.75-0.5:1) 5(<0.5:1))  Note: when standardising, 3 was treated as 0 point, and 1 and 5 were treated a 1 (highest impact). |
