# Supplementary material for: Assessing Variation in the Individual-Level Impacts of a Multihost Pathogen
Source: Transbound Emerg Dis. 2023 May 27;2023:4003285. doi: 10.1155/2023/4003285 (PMC12017245; doi:10.1155/2023/4003285)
Supplement: Supplementary Materials — S1: Excel file containing data frames used in data collection and analysis for this project, as well as updated host species database for Sarcoptes scabiei. These data are deposited in the UTAS Research Data Portal repository (https://doi.org/10.25959/12q1-g388). S2: species inclusion criteria for the study. Table S3: the grading criteria used, and the assigned number of intervals, for quantifying and standardising each individual-level pathogen impact that formed the AIS. Table S4: confidence in standardisation criteria: required interpretation needed to quantify pathogen impacts from the literature. Figure S5: the initial rank of host species plotted against the average impact score (AIS), with the AIS range shown in error bars. Plotted to the right: average sample size per impact (log + 1), total pathogen impacts assessed per species (in light grey, impacts that were standardised into binary data, and in dark grey, impacts standardised into interval data). Table S6: Pearson correlation matrix for continuous predictor variables is used in regression analysis. Significant values (R > 0.7) are in bold. Figure S7: diagnostic plots for inclusion cut offs (vertical red lines) for the conservative rank of Sarcoptes scabiei impacts among host species, showing average impact score for all host species in initial rank (77) against (A) average sample size per impact (log + 1) (with cut off at 4), (B) average confidence in standardisation per impact scores (with cut off at 2.5), (C) total pathogen impacts assessed per species (with cut off at 4), and (D) studies used per species (with cut off at 2), refer to Table 1 for justification of cut off values. Table S8: the table showing the proportion of families and species known to be infected by Sarcoptes scabiei for each affected order. Figure S9: boxplot of host species from conservative AIS rank organised into their taxonomic family plotted against average impact score. Red numbers represent the number of species in each fami [file 4003285.f1.zip › Supplementary Material 5 (1).docx]

**
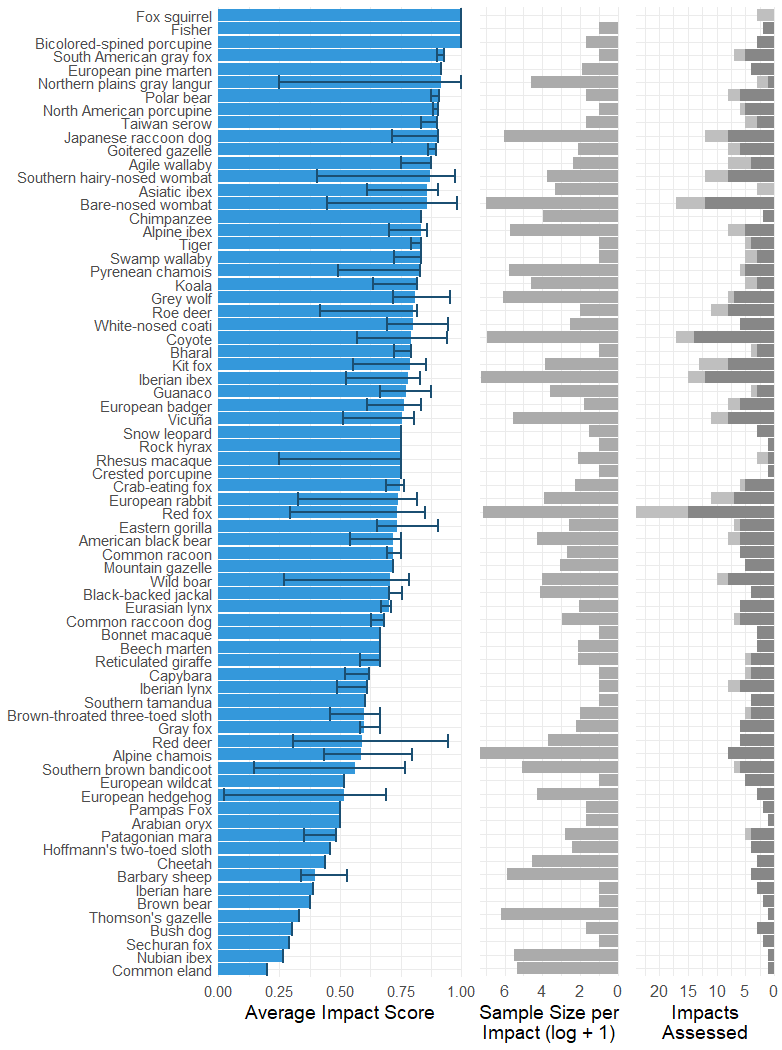
S5**. The initial rank of host species, plotted against Average Impact Score (AIS), with the AIS range shown in error bars. Plotted to the right: Average Sample Size per Impact (log + 1), Total pathogen impacts assessed per species (in light grey, impacts that were standardised into binary data, and in dark grey, impacts standardised into interval data).
